# Segregated Patterns of Hospital Care Delivery and Health Outcomes

**DOI:** 10.1001/jamahealthforum.2023.4172

**Published:** 2023-11-22

**Authors:** Sunny C. Lin, Gmerice Hammond, Michael Esposito, Cassandra Majewski, Randi E. Foraker, Karen E. Joynt Maddox

**Affiliations:** 1 Division of General Medical Sciences, Washington University School of Medicine in St Louis, St Louis, Missouri; 2 Institute for Informatics, Washington University in St Louis, St Louis, Missouri; 3 Institute for Public Health, Washington University in St Louis, St Louis, Missouri; 4 Cardiovascular Division, Washington University School of Medicine in St Louis, St Louis, Missouri; 5 Department of Sociology, University of Minnesota, Minneapolis

## Abstract

**Importance:**

Residential segregation has been shown to be a root cause of racial inequities in health outcomes, yet little is known about current patterns of racial segregation in where patients receive hospital care or whether hospital segregation is associated with health outcomes. Filling this knowledge gap is critical to implementing policies that improve racial equity in health care.

**Objective:**

To characterize contemporary patterns of racial segregation in hospital care delivery, identify market-level correlates, and determine the association between hospital segregation and health outcomes.

**Design, Setting, and Participants:**

This cross-sectional study of US hospital referral regions (HRRs) used 2018 Medicare claims, American Community Survey, and Agency for Healthcare Research and Quality Social Determinants of Health data. Hospitalization patterns for all non-Hispanic Black or non-Hispanic White Medicare fee-for-service beneficiaries with at least 1 inpatient hospitalization in an eligible hospital were evaluated for hospital segregation and associated health outcomes at the HRR level. The data analysis was performed between August 10, 2022, and September 6, 2023.

**Exposures:**

Dissimilarity index and isolation index for HRRs.

**Main Outcomes and Measures:**

Health outcomes were measured using Prevention Quality Indicator (PQI) acute and chronic composites per 100 000 Medicare beneficiaries, and total deaths related to heart disease and stroke per 100 000 residents were calculated for individuals aged 74 years or younger. Correlation coefficients were used to compare residential and hospital dissimilarity and residential and hospital isolation. Linear regression was used to examine the association between hospital segregation and health outcomes.

**Results:**

This study included 280 HRRs containing data for 4386 short-term acute care and critical access hospitals. Black and White patients tended to receive care at different hospitals, with a mean (SD) dissimilarity index of 23 (11) and mean (SD) isolation index of 13 (13), indicating substantial variation in segregation across HRRs. Hospital segregation was correlated with residential segregation (correlation coefficients, 0.58 and 0.90 for dissimilarity and isolation, respectively). For Black patients, a 1-SD increase in the hospital isolation index was associated with 204 (95% CI, 154-254) more acute PQI hospitalizations per 100 000 Medicare beneficiaries (28% increase from the median), 684 (95% CI, 488-880) more chronic PQI hospitalizations per 100 000 Medicare beneficiaries (15% increase), and 6 (95% CI, 2-9) additional deaths per 100 000 residents (6% increase) compared with 68 (95% CI, 24-113; 6% increase), 202 (95% CI, 131-274; 8% increase), and 2 (95% CI, 0 to 4; 3% increase), respectively, for White patients.

**Conclusions and Relevance:**

This cross-sectional study found that higher segregation of hospital care was associated with poorer health outcomes for both Black and White Medicare beneficiaries, with significantly greater negative health outcomes for Black populations, supporting racial segregation as a root cause of health disparities. Policymakers and clinical leaders could address this important public health issue through payment reform efforts and expansion of health insurance coverage, in addition to supporting upstream efforts to reduce racial segregation in hospital care and residential settings.

## Key Points

**Question:**

To what extent are hospital visits for Medicare beneficiaries sorted into different hospitals by race, and how is this measure of racial segregation associated with health outcomes?

**Findings:**

In this cross-sectional study of Medicare claims data for 4386 hospitals across 280 hospital referral regions, in many regions, Black and White Medicare beneficiaries received care at different hospitals in correlation with residential segregation. Hospital segregation was associated with worse health outcomes for both Black and White populations as well as with greater racial differences in health outcomes.

**Meaning:**

The findings suggest that in many hospital markets, Black and White Medicare beneficiaries visit different hospitals; thus, policies that address racial segregation could improve health outcomes and racial equity in health care.

## Teaser

This cross-sectional study characterizes patterns of racial segregation among Medicare beneficiaries in hospital care delivery and evaluates their association with health outcomes.

## Introduction

Racial inequities in health outcomes are a public health crisis in the US, and segregation is a root cause of this inequity.^1^ The origins of segregation are deep in the US, a country founded on belief systems that upheld the enslavement of Black individuals and genocide of American Indian communities. Since then, racist belief systems have been encoded into US policies that dictate where and how individuals can live. These policies have led to the residential segregation of Black communities into geographic areas that not only are subjected to greater environmental and contextual harms but also have fewer resources, such as health care, to mitigate the impact of those harms.^2,3^ Although policies promoting residential segregation on the basis of race and ethnicity were formally outlawed in the 1960s, informal practices and the harmful effects of segregation have persisted. Residential segregation is associated with exposure to health risks, such as contaminated water, urban heat islands, police violence, predatory lending, food deserts, and more.^3^

However, less is known about contemporary levels or effects of hospital segregation, defined in this article as the degree to which patients receive care at different hospitals within the same market based on their race. In the US, many hospitals refused to care for Black patients until after 1966 when the creation of Medicare enabled the federal government to withhold funding to hospitals that practiced racial discrimination.^4,5,6^ In 1956, nearly 1 in 3 hospitals in southern states did not admit Black patients under any condition, including emergencies.^7^ Anecdotally, health care segregation remains, especially in major metropolitan areas, such as New York City and Boston. However, some studies have found that health care is relatively desegregated, especially compared with other services, such as public education.^8,9^ Prior work has suggested that factors such as differential access to care, physician referral patterns, and community experiences of discrimination may reinforce historical patterns of hospital segregation,^9,10,11^ whereas other factors, such as expanded insurance coverage, may help to desegregate hospitals.

To our knowledge, there has been no empirical national examination of hospital segregation, its health consequences, or factors that contribute to or protect against it in the US since 1993.^8^ There are compelling reasons to be concerned about the contemporary state of hospital segregation in the US. Since the 1990s, Black and White segregation has increased across educational and residential settings,^12,13^ the wealth gap between Black and White individuals is widening,^14^ and the improvement in the disparity between Black and White population-level mortality has stagnated.^15^

Understanding the state of racial segregation across US hospitals is crucial for improving health equity. The aim of this study was to describe contemporary patterns of hospital segregation within hospital markets in the US, identify local market-level correlates of hospital segregation, and examine the association between hospital segregation and health outcomes. For this study, we chose to focus specifically on non-Hispanic Black and non-Hispanic White segregation due to long-standing and deeply rooted anti-Black racism in the US that has and continues to impose a more rigid and persistent divide between Black and White groups than other racialized groups.^16,17^

## Methods

### Data Sources and Study Population

The population of this cross-sectional study included all Medicare patients hospitalized in a short-term acute care hospital or critical access hospital in the US between January 1 and December 31, 2018. We combined 100% Medicare inpatient fee-for-service claims files with data from the Dartmouth Atlas and the Agency for Healthcare Research and Quality’s Social Determinants of Health database. This study was approved by the Office of Human Research Protection at Washington University in St Louis, which waived the requirement for informed consent due to the deidentified nature of the data. We followed the Strengthening the Reporting of Observational Studies in Epidemiology (STROBE) guideline for reporting cross-sectional studies.

### Racial Segregation Measures

We calculated 2 measures of racial segregation within hospital referral regions (HRRs): the dissimilarity index and the isolation index. Segregation was calculated at the area level (HRR) rather than at the hospital level because the area level examines how patients sort between hospitals rather than within hospitals. Hospital referral regions are groups of zip codes based on where residents in a region seek most of their hospital care.^18^ We chose HRRs as our unit of analysis since they capture patterns of hospital care better than geopolitical boundaries, such as counties or states, and we chose HRRs over hospital service areas since many hospital service areas contain only 1 hospital (rendering between-hospital segregation incalculable).^19^

Both the dissimilarity index and the isolation index are commonly used in the literature on residential segregation and measure different and complementary dimensions.^8,9,20,21^ They range from 0 (no segregation) to 100 (complete segregation). The dissimilarity index can be interpreted as the percentage of individuals who would have to move from 1 unit (ie, hospital or zip code tabulation area [ZCTA]) to another unit within the HRR to make racial composition across all units even. The isolation index can be interpreted as the probability of an average Black resident sharing a unit (hospital or ZCTA) with another Black resident. The dissimilarity measure is a high-level view of how evenly distributed the racial groups are across a geographic area; importantly, this measure does not account for the relative sizes of the groups being compared. Thus, it allows us to see how racial segregation can manifest in areas with a low proportion of Black residents. The isolation measure is more reflective of the average person’s experience and takes into account the relative size of the groups being compared.^22^

We calculated residential and hospital segregation measures within HRRs using analogous methods. The racial composition of residents in each ZCTA was obtained from the 5-year estimates of the American Community Survey (ACS) for 2016 through 2020. Racial categories were self-selected by respondents to the ACS. The racial composition of patients in each hospital was obtained from 2018 Medicare inpatient data, and thus we included only hospitals that admitted at least 1 Medicare patient in 2018. Racial categories in Medicare were obtained from the Research Triangle Institute–imputed race variable derived from the Social Security database.^23^

### Market Covariates

We examined HRR-level characteristics that we hypothesized to be associated with hospital segregation and health care inequities based on a conceptual model built on a review of the literature on racial segregation and health care access (eFigure 1 in Supplement 1).^24^ These characteristics included percentage of noncitizens, median age, median income, difference in median income for Black compared with White residents, percentage of Black residents living in poverty, percentage of White residents living in poverty, percentage of adult residents with less than a high school education, percentage of the population aged 64 years or younger with any Medicaid or other means-tested public health insurance coverage, percentage of the population with Medicare only, percentage of the population with employer-based health insurance, total number of hospitals, percentage of Black residents, total population, percentage of residents living in an urban (metropolitan) county, and geographic region based on Census Bureau regions (ie, Midwest, Northeast, South, and West), each calculated at the HRR level by taking the population-weighted average of all ZCTAs in the HRR. Zip code tabulation areas were mapped to zip codes using a crosswalk from the census and then to HRRs using a crosswalk from the Dartmouth Atlas.

### Health Outcome Measures

We compared health outcomes using 3 measures from the 2019 Agency for Healthcare Research and Quality’s Social Determinants of Health database: (1) the Prevention Quality Indicator (PQI) acute composite, (2) the PQI chronic composite, and (3) total heart disease and stroke deaths per 100 000 residents aged 74 years or younger. These measures were publicly available by race at the county level and averaged to the HRR level. The PQI acute composite measures potentially preventable hospitalizations for dehydration, bacterial pneumonia, or urinary tract infection. The PQI chronic composite measures potentially preventable hospitalizations for diabetes with short-term complications, diabetes with long-term complications, uncontrolled diabetes, diabetes with lower-extremity amputation, chronic obstructive pulmonary disease, asthma, hypertension, heart failure, or angina without a cardiac procedure.

### Statistical Analysis

The data analysis was performed between August 10, 2022, and September 6, 2023. After calculating our residential and hospital segregation measures, we compared the 2 by calculating the correlation coefficients between residential and hospital dissimilarity and residential and hospital isolation. To understand how market characteristics were associated with hospital segregation, we ran multiple linear regression models using market characteristics to estimate hospital segregation. To examine the association between hospital segregation and health outcomes, we ran unadjusted and adjusted linear regression models. We ran 3 sets of models per outcome: (1) for Black populations, (2) for White populations, and (3) for the difference between the 2 populations. To illustrate unadjusted associations, we generated scatterplots of outcomes for Black and White populations by segregation. For the adjusted models, we included all market covariates listed above. Because of collinearity, we did not include other measures of segregation in the adjusted models (eg, in the model with hospital dissimilarity, we did not include residential isolation, residential dissimilarity, or hospital isolation). For the same reason, we also dropped the percentage of Black residents in our models examining residential and hospital isolation. For ease of interpretation, we standardized our main outcome (segregation) in both adjusted and unadjusted models and estimated the association between a 1-SD increase in segregation and outcomes. For sensitivity analyses, we reran our models using weights for HRR residential population to provide results that can be interpreted for US population-level estimates. We also ran a sensitivity analysis with regional random effects for each covariate to allow for market characteristics to influence hospital segregation differently by region. Analyses were conducted using Stata, version 17.0 statistical software (StataCorp LLC). All tests were conducted using a 2-sided *P* < .05.

## Results

**Figure 1.**
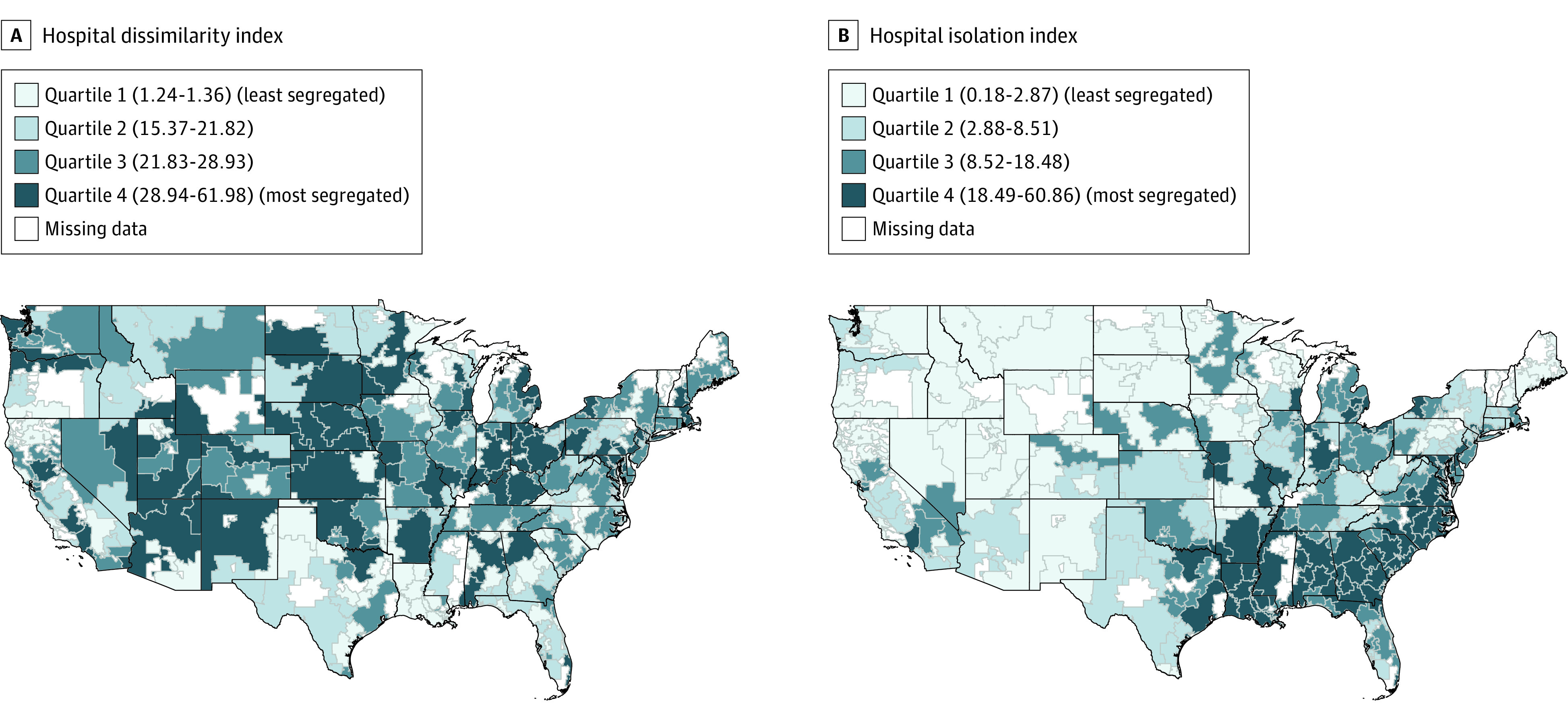
Segregated Patterns of Hospital Care Delivery Between Black and White Medicare Patients Within US Hospital Referral Regions Hospital segregation is calculated at the hospital referral region (n = 280) level to show how patterns of hospital visits are segregated by race between hospitals.

After matching hospitals in the Medicare claims data to market characteristics, our final analytic data set included 4386 hospitals across 280 HRRs; of these, 4 HRRs with a low number of Black residents were missing data on death rates for Black patients and were dropped from the models of death rates (eFigure 2 in Supplement 1). We present the 5 most and least segregated HRRs among the 40 biggest HRRs by population in Table 1. The mean (SD) level of hospital dissimilarity was 23 (11), the mean (SD) level of hospital isolation was 13 (13), the mean (SD) level of residential dissimilarity was 40 (12), and the mean (SD) level of residential isolation was 21 (17). Maps of the hospital dissimilarity and isolation indices across HRRs are shown in Figure 1.

**Table 1.** Most and Least Segregated Hospital Referral Regions (HRRs) of the Top 40 Biggest HRRs by Population^a^

HRR name	Health care dissimilarity index or hospital isolation index	Residential dissimilarity index or residential isolation index	Black population, millions	Total population, millions	No. of hospitals	Region
**Top 5 most segregated**						
Hospital dissimilarity						
Washington, District of Columbia	55	50	3.8	10.3	20	South
Detroit, Michigan	51	71	0.7	2.0	14	Midwest
St Louis, Missouri	50	67	0.7	4.5	59	Midwest
Indianapolis, Indiana	49	55	0.4	4.3	50	Midwest
Minneapolis, Minnesota	45	49	0.3	3.9	70	Midwest
Hospital isolation						
Detroit, Michigan	60	73	0.7	2.0	14	Midwest
Washington, District of Columbia	57	58	3.8	10.3	20	South
Chicago, Illinois	54	64	0.8	2.9	26	Midwest
Memphis, Tennessee	41	65	1.6	3.4	25	South
Baltimore, Maryland	40	56	1.2	3.6	24	South
**Bottom 5 least segregated**						
Hospital dissimilarity						
San Antonio, Texas	21	41	0.2	4.3	29	South
Orlando, Florida	22	38	1.1	7.5	31	South
Tulsa, Oklahoma	23	49	0.2	2.2	38	South
Houston, Texas	23	38	2.1	12.3	75	South
Charlotte, North Carolina	23	41	1.5	5.2	20	South
Hospital isolation						
Lexington, Kentucky	5	18	0.3	3.6	40	South
Phoenix, Arizona	6	9	0.3	6.1	41	West
San Antonio, Texas	7	11	0.2	4.3	29	South
Seattle, Washington	7	11	0.2	4.6	24	West
Albany, New York	8	26	0.2	2.7	25	Northeast

^a^ Measures of hospital segregation are based on the number of Medicare visits to area hospitals. Residential segregation is based on the number of residents living in area zip codes.

### Factors Associated With Hospital Segregation

The correlation coefficients between hospital and residential segregation were 0.58 (95% CI, 0.88-0.92) for dissimilarity and 0.90 (95% CI, 0.50-0.65) for isolation (eTable 1 and eFigure 3 in Supplement 1). The hospital dissimilarity index was higher in regions with higher residential segregation, with higher median income, with more hospitals, with a larger percentage of rural residents, and in the Midwest compared with the Northeast and South (Table 2). The hospital isolation index was higher in regions with higher residential segregation, a greater percentage of noncitizens, a greater percentage of White residents living in poverty, a lower percentage of Medicare and employer-insured beneficiaries, a greater percentage of Black residents, and a larger number of residents, as well as in the Midwest compared with the Northeast and South (Table 2). Fully adjusted regression results are presented in eTables 2 to 4 in Supplement 1.

**Table 2.** Multiple Linear Regression Results for Factors Associated With Hospital Segregation in 280 Hospital Referral Regions

Factor	Coefficient (95% CI)	
	Hospital dissimilarity	Hospital isolation
Residential dissimilarity	0.45 (0.36 to 0.55)^a^	NA
Residential isolation	NA	0.35 (0.26 to 0.44)^a^
Percentage noncitizen	0.01 (−0.39 to 0.41)	0.25 (0.00 to 0.49)^b^
Median age	0.07 (−0.43 to 0.57)	0.26 (−0.04 to 0.57)
Median income, $1000s	2.04 (0.48 to 3.60)^b^	0.66 (−0.30 to 1.62)
Difference in Black and White income, $1000s	0.35 (−0.70 to 1.41)	0.37 (−0.28 to 1.02)
Percentage living in poverty		
Black	−0.18 (−0.39 to 0.03)	−0.13 (−0.25 to 0.00)
White	0.35 (−0.06 to 0.77)	0.36 (0.11 to 0.62)^c^
Percentage with less than high school education	0.02 (−0.39 to 0.44)	0.13 (−0.12 to 0.39)
Percentage with Medicaid	−0.07 (−0.40 to 0.25)	−0.12 (−0.32 to 0.08)
Percentage with Medicare	−0.13 (−1.26 to 1.00)	−0.90 (−1.60 to −0.21)^b^
Percentage employer insured	−1.99 (−5.31 to 1.34)	−2.05 (−4.09 to −0.01)^b^
No. of hospitals	0.23 (0.12 to 0.33)^a^	−0.04 (−0.10 to 0.03)
Percentage of Black residents	0.02 (−0.09 to 0.12)	0.56 (0.43 to 0.70)^a^
No. of residents, millions	0.57 (−0.15 to 1.29)	0.62 (0.18 to 1.07)^c^
Percentage of population living in urban counties	−0.08 (−0.14 to −0.01)^b^	−0.01 (−0.05 to 0.03)
Region (reference, Midwest)		
Northeast	−3.71 (−6.84 to −0.58)^b^	−3.34 (−5.28 to −1.40)^a^
South	−3.79 (−6.94 to −0.64)^b^	−2.94 (−4.87 to −1.01)^c^
West	−0.43 (−3.97 to 3.11)	−0.67 (−2.86 to 1.52)
Constant	−2.52 (−26.48 to 21.44)	−8.37 (−23.14 to 6.40)
*R* ^2^	0.616	0.889

Abbreviation: NA, not applicable.

^a^ *P* < .001.

^b^ *P* < .05.

^c^ *P* < .01.

### Hospital Segregation and Health Outcomes

**Figure 2.**
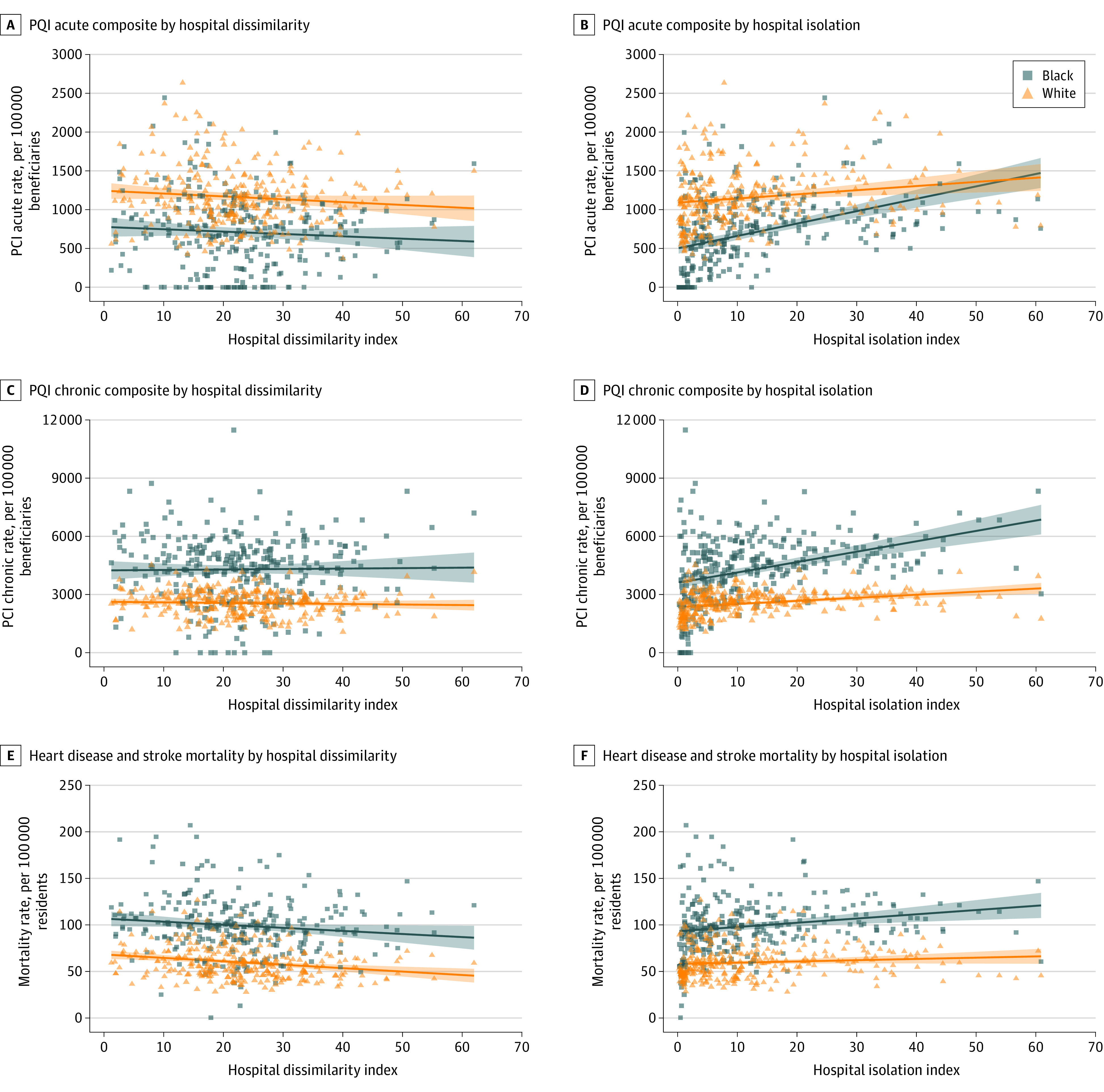
Unadjusted Associations Between Health Outcomes and Hospital Segregation Lines represent linear trends and shaded areas the 95% CIs for outcomes by segregation. Hospital segregation is calculated at the hospital referral region (n = 280) level to show how patterns of hospital visits are segregated by race between hospitals. PQI indicates Prevention Quality Indicator.

A higher hospital dissimilarity index was associated with lower mortality rates for both Black and White populations in unadjusted analyses (Figure 2). After adjusting for market covariates, this association was attenuated and no longer statistically significant (Table 3). A higher hospital isolation index was associated with worse outcomes for both racial groups and greater racial disparities for all 3 outcomes (Table 3). In unadjusted analysis, a 1-SD increase in the hospital isolation index was associated with an increase in the PQI acute composite of 204 (95% CI, 154-254) admissions per 100 000 Black Medicare beneficiaries and 68 (95% CI, 24-113) per 100 000 White Medicare beneficiaries, a 28% and 6% increase from their respective medians; an increase in the PQI chronic composite of 684 (95% CI, 488-880) admissions per 100 000 Black Medicare beneficiaries and 202 (95% CI, 131-274) per 100 000 White Medicare beneficiaries, a 15% and 8% increase from their respective medians; and an increase in deaths related to heart disease or stroke of 6 (95% CI, 2-9) per 100 000 Black residents and 2 (95% CI, 0-4) per 100 000 White residents, a 6% and 3% increase from their respective medians. After adjusting for market covariates, the association between the hospital isolation index and racial disparities in outcomes was attenuated but remained statistically significant (Table 3).

**Table 3.** Standardized Associations Between Hospital Referral Region (HRR)–Level Hospital Segregation and Health Outcomes

	PQI acute composite per 100 000 Medicare beneficiaries		PQI chronic composite per 100 000 Medicare beneficiaries		Heart disease or stroke deaths per 100 000 residents	
	Unadjusted	Adjusted	Unadjusted	Adjusted	Unadjusted	Adjusted
**HRR level, median (IQR)** ^a^						
Black populations	732 (368 to 986)	NA	4535 (3221 to 5480)	NA	98 (84 to 113)	NA
White populations	1107 (905 to 1348)	NA	2632 (2132 to 2973)	NA	58 (47 to 70)	NA
Difference between populations	−392 (−691 to −127)	NA	1923 (1007 to 2656)	NA	38 (28 to 49)	NA
**Regression results, coefficient (95% CI)**						
Hospital dissimilarity						
Black populations	−34 (−89 to 21)	−10 (−75 to 54)	26 (−186 to 238)	107 (−143 to 358)	−4 (−7 to 0)^b^	−3 (−8 to 1)
White populations	−41 (−86 to 4)	−15 (−61 to 32)	−31 (−107 to 44)	35 (−40 to 109)	−4 (−6 to −2)^c^	−1 (−2 to 1)
Difference	7 (−51 to 65)	5 (−59 to 69)	57 (−127 to 241)	73 (−156 to 301)	1 (−2 to 3)	−3 (−6 to 1)
Hospital isolation						
Black populations	204 (154 to 254)^c^	157 (94 to 220)^c^	684 (488 to 880)^c^	343 (101 to 584)^d^	6 (2 to 9)^d^	4 (0 to 8)^b^
White populations	68 (24 to 113)^d^	15 (−30 to 60)	202 (131 to 274)^c^	65 (−7 to 137)	2 (0 to 4)	−2 (−4 to 0)^b^
Difference	136 (80 to 192)^c^	142 (80 to 204)^c^	481 (307 to 656)^c^	277 (57 to 498)^b^	4 (2 to 7)^d^	6 (2 to 9)^d^
Residential dissimilarity						
Black populations	−3 (−58 to 53)	−49 (−111 to 12)	315 (106 to 524)^d^	131 (−107 to 370)	0 (−4 to 3)	−1 (−5 to 3)
White populations	−8 (−53 to 37)	−59 (−103 to −15)^d^	133 (59 to 207)^c^	−3 (−74 to 69)	−1 (−3 to 1)	0 (−1 to 2)
Difference	6 (−52 to 64)	10 (−51 to 70)	182 (−1 to 365)	134 (−83 to 351)	1 (−2 to 4)	−2 (−5 to 2)
Residential isolation						
Black populations	216 (166 to 265)^c^	177 (112 to 243)^c^	720 (525 to 914)^c^	381 (128 to 633)^d^	6 (3 to 10)^c^	4 (−1 to 8)
White populations	105 (61 to 148)^c^	11 (−36.3 to 58)	265 (197 to 334)^c^	68 (−8 to 143)	3 (1 to 5)^d^	−1 (−3 to 1)
Difference	111 (55 to 167)^c^	166 (102 to 230)^c^	454 (278 to 630)^c^	313 (83 to 544)^d^	3 (0 to 6)^b^	4 (0 to 8)^b^

Abbreviations: NA, not applicable; PQI, Prevention Quality Indicator.

^a^ Adjusted models include percentage of noncitizens, median age, median income, difference in median income for Black compared with White residents, percentage of Black residents living in poverty, percentage of White residents living in poverty, percentage of adult residents with less than a high school education, percentage of the population aged 64 years or younger with Medicaid or other public health insurance, percentage of the population with Medicare only, percentage of the population with employer-based health insurance, total number of hospitals, number of residents, percentage of the population living in an urban county, and geographic region; the adjusted model for dissimilarity measures includes percentage of Black residents as an additional control variable. Sum may not total to differences due to rounding.

^b^ *P* < .05.

^c^ *P* < .001.

^d^ *P* < .01.

### Sensitivity Analyses

Results from the models with regional-level random effects (eTable 5 in Supplement 1) were similar to the main analysis. Weights for the number of residents per HRR (eTables 6 and 7 in Supplement 1) also were similar to those of the main analyses.

## Discussion

In this cross-sectional study, we characterized the extent of racial segregation between US hospitals within HRRs and its association with health outcomes using 2 separate dimensions of segregation. We found that segregation in hospital care was associated with residential segregation, suggesting that the 2 may be inextricably linked. We also found that higher levels of segregation in hospital care were associated with poorer outcomes for both racial groups, with a greater negative association for Black populations.

Our finding that residential and hospital segregation may be tightly linked supports the notion that residential segregation not only exposes racial and ethnic minority groups to more harmful environmental and contextual conditions but also limits these groups’ access to high-quality health care services with which to mitigate that harm.^2^ Racial segregation in hospital settings may be explained by the lack of intentional policy efforts to actively address structural, upstream root causes of hospital segregation. Instead, recent efforts to address racism in health care have tended to focus on interpersonal discrimination, such as implicit bias and medical training.^25^

Our finding that residential segregation and insurance coverage are associated with hospital segregation also suggests that addressing upstream determinants of hospital segregation (eg, expanding public and private insurance coverage) may be effective in helping to desegregate hospital settings. In addition, these upstream factors could create more equitable funding mechanisms to support health care professionals and organizations who serve racially segregated Black communities. Other support mechanisms could come in the form of increased funding to prevent hospital closures, supporting the training and advancement of Black physicians, and implementing need-based reimbursement models.^26^ In addition, we found that market-level factors associated with isolation and dissimilarity indices were different and that the isolation index was more closely associated with outcomes than the dissimilarity index. These findings suggest that technical decisions on how to measure racial segregation should be made thoughtfully, keeping in mind the implications and underlying processes that contribute to each measure. For example, the isolation index was more sensitive to the overall proportion of Black residents in an area. This finding suggests that broad, historical patterns of Black migration may have a strong intergenerational impact on hospital segregation.^21^ Along these lines, future research should incorporate historical place-based policies and structures into the study of hospital segregation.^27^

Our findings also suggest that variation in racial segregation across US hospital markets may lead to inequitable outcomes from health reform efforts.^8^ For example, previous work has found that hospitals that participate in payment reform efforts may disproportionately serve affluent White communities.^28,29,30,31^ Explicit and thoughtful approaches to health care reform must acknowledge that hospitals do not operate in a vacuum. The equity impact of health care reform programs may be influenced by the broader hospital context, including racial segregation in the market. For example, Medicare’s Shared Savings Program evaluates organizational success by comparing the performance of health care organizations with their neighbors.^32^ In segregated areas, this comparison may shift resources away from Black communities, exacerbating racial inequities caused by segregation. As Medicare aims to move reimbursements to value-based payment,^33^ it is imperative that the Centers for Medicare & Medicaid Services continually monitor program results and adjust program design in order to direct resources into, and not away from, communities where resources are most needed.^34^ Other policies could combat the ill effects of racial segregation by providing stronger incentives for hospitals to address community needs, including making a more intentional effort to expand access.^35^ For example, *US News & World Report* recently piloted a set of racial equity measures that reflect areas where hospitals have the opportunity to improve health care quality for the most vulnerable patients in their community.^36^ Medicare’s new Accountable Care Organization Realizing Equity, Access, and Community Health program attempts to address equity by providing financial incentives for organizations to create and implement a community-based health equity plan. Integrating equity measures into payment reform efforts may also provide a unique opportunity to improve health care equity.

Our work should be interpreted in the context of related literature. In 1998, Smith^8^ reported that the mean dissimilarity index for hospital segregation among Black and White Medicare beneficiaries across US states was 53, and in 2009, Sarrazin et al^9^ found that the mean dissimilarity index and isolation index among Medicare beneficiaries admitted for acute myocardial infarction in 2004-2005 were 37 and 22, respectively, among 105 HRRs. While results from this study cannot be directly compared with these analyses due to differences in methodology, our results are consistent with this prior work that finds that racial segregation in health care delivery is persistent, even though formal practices of racial segregation have been prohibited for decades.^4,8,9,37^ Furthermore, our finding that segregation is associated with racial disparities in outcomes is consistent with prior research showing that Black surgical patients may be more likely to receive care from lower-quality providers.^28,38,39,40^

### Limitations

Our study has several limitations. First, our measure of segregation is based on patterns of hospital care for Medicare beneficiaries, a fully insured population, and thus may underestimate care segregation. Patterns of segregation may be different for other groups and more sensitive to racial inequities in health care access. In addition, our measure is based on patterns of hospital care, not ambulatory or long-term care, which may be more segregated. Second, ACS data on race and ethnicity for this study were obtained at the ZCTA level, which are geographic areas designated by the US Census Bureau that approximate zip code areas. Thus, some residents may have been double-counted when ZCTA data were aggregated to the HRR level. However, because data on racial composition are not available at the zip code level, ZCTA codes are the best-available approximation of HRR-level racial composition. In addition, this methodology is most directly comparable with prior work on residential segregation at the HRR level.^9,38^ Furthermore, since race is a social construct, measures of race are contextual and subjective and may not adequately capture the experiences or identities of individuals.^41^

A third limitation is that this study focuses exclusively on racial segregation between non-Hispanic Black and non-Hispanic White populations, and results are not generalizable to other racialized groups. Future work should examine how hospital segregation differs for other racialized groups. The current 6-category Office of Management and Budget standard for the collection of race and ethnicity data also masks important subgroup differences, especially among Asian and Hispanic panethnic groups. Owing to different histories and patterns of immigration, disaggregated race and ethnicity data for these groups are critical in the study of hospital segregation and outcomes for other racialized populations.^42^ Finally, health care segregation can manifest in many ways. Our study conceptualizes hospital segregation as the sorting of patients into different hospital systems based on race, which captures only 1 facet of segregation. For example, our measures do not provide insight into how segregation manifests within hospitals, which prior work suggests may also have a profound influence on health equity.^39^

## Conclusions

This cross-sectional study’s findings suggest that hospital care remains segregated by race, with Black and White patients receiving care at different hospitals within the same hospital markets. This type of segregation is associated with substantial disparities in health outcomes. Policymakers and clinical leaders should consider policy solutions to address this important public health issue, such as incorporating evaluations of racial equity in payment reform efforts and expanding health insurance coverage to reduce racial segregation in hospital care.

## Notes

Supplement 1. **eFigure 1.** Conceptual Model **eFigure 2.** Study Sample **eFigure 3.** Scatter Plots of Residential and Hospital Segregation Across US Hospital Referral Regions **eTable 1.** Correlation Matrix: Residential and Hospital Segregation **eTable 2.** Fully Adjusted Regression Results: PQI Acute Composite **eTable 3.** Fully Adjusted Regression Results: PQI Chronic Composite **eTable 4.** Fully Adjusted Regression Results: Heart Disease or Stroke Deaths **eTable 5.** Sensitivity Analysis, Regional Random Effects **eTable 6.** Sensitivity Analysis, Weighted by HRR Population: Multiple Linear Regression Results, Predictors of Hospital Segregation **eTable 7.** Sensitivity Analysis, Weighted by HRR Resident Population: Standardized Associations Between Hospital Segregation and Health Outcomes

Supplement 2.
Data Sharing Statement
