# Supplementary material for: Segregated Patterns of Hospital Care Delivery and Health Outcomes
Source: JAMA Health Forum. 2023 Nov 22;4(11):e234172. doi: 10.1001/jamahealthforum.2023.4172 (PMC10665978; doi:10.1001/jamahealthforum.2023.4172)
Supplement: Supplement 1. — eFigure 1. Conceptual Model eFigure 2. Study Sample eFigure 3. Scatter Plots of Residential and Hospital Segregation Across US Hospital Referral Regions eTable 1. Correlation Matrix: Residential and Hospital Segregation eTable 2. Fully Adjusted Regression Results: PQI Acute Composite eTable 3. Fully Adjusted Regression Results: PQI Chronic Composite eTable 4. Fully Adjusted Regression Results: Heart Disease or Stroke Deaths eTable 5. Sensitivity Analysis, Regional Random Effects eTable 6. Sensitivity Analysis, Weighted by HRR Population: Multiple Linear Regression Results, Predictors of Hospital Segregation eTable 7. Sensitivity Analysis, Weighted by HRR Resident Population: Standardized Associations Between Hospital Segregation and Health Outcomes [file jamahealthforum-e234172-s001.pdf]

Supplemental Online Content

Lin SC, Hammond G, Esposito M, Majewski C, Foraker R, Joynt Maddox KE. Segregated patterns of hospital care delivery and health outcomes. *JAMA Health Forum*. 2023;4(11):e234172. doi:10.1001/jamahealthforum.2023.4172

eFigure 1. Conceptual Model

eFigure 2. Study Sample

eFigure 3. Scatter Plots of Residential and Hospital Segregation Across US Hospital Referral Regions

eTable 1. Correlation Matrix: Residential and Hospital Segregation

eTable 2. Fully Adjusted Regression Results: PQI Acute Composite

eTable 3. Fully Adjusted Regression Results: PQI Chronic Composite

eTable 4. Fully Adjusted Regression Results: Heart Disease or Stroke Deaths

eTable 5. Sensitivity Analysis, Regional Random Effects

eTable 6. Sensitivity Analysis, Weighted by HRR Population: Multiple Linear Regression Results, Predictors of Hospital Segregation

eTable 7. Sensitivity Analysis, Weighted by HRR Resident Population: Standardized Associations Between Hospital Segregation and Health Outcomes

This supplemental material has been provided by the authors to give readers additional information about their work.

© 2023 Lin SC et al. *JAMA Health Forum.*

eFigure 1. Conceptual Model

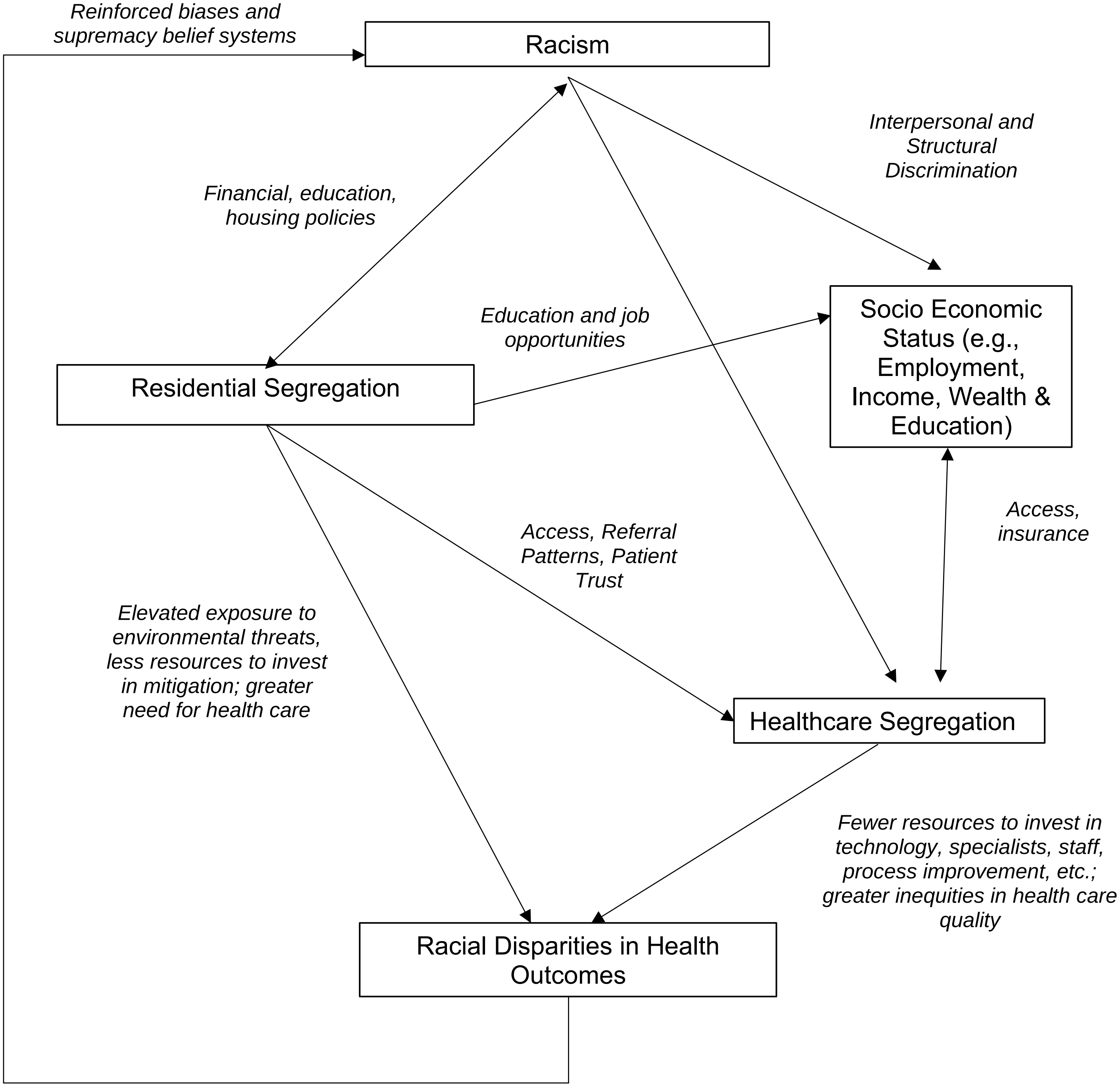

© 2023 Lin SC et al. *JAMA Health Forum.*

eFigure 2. Study Sample

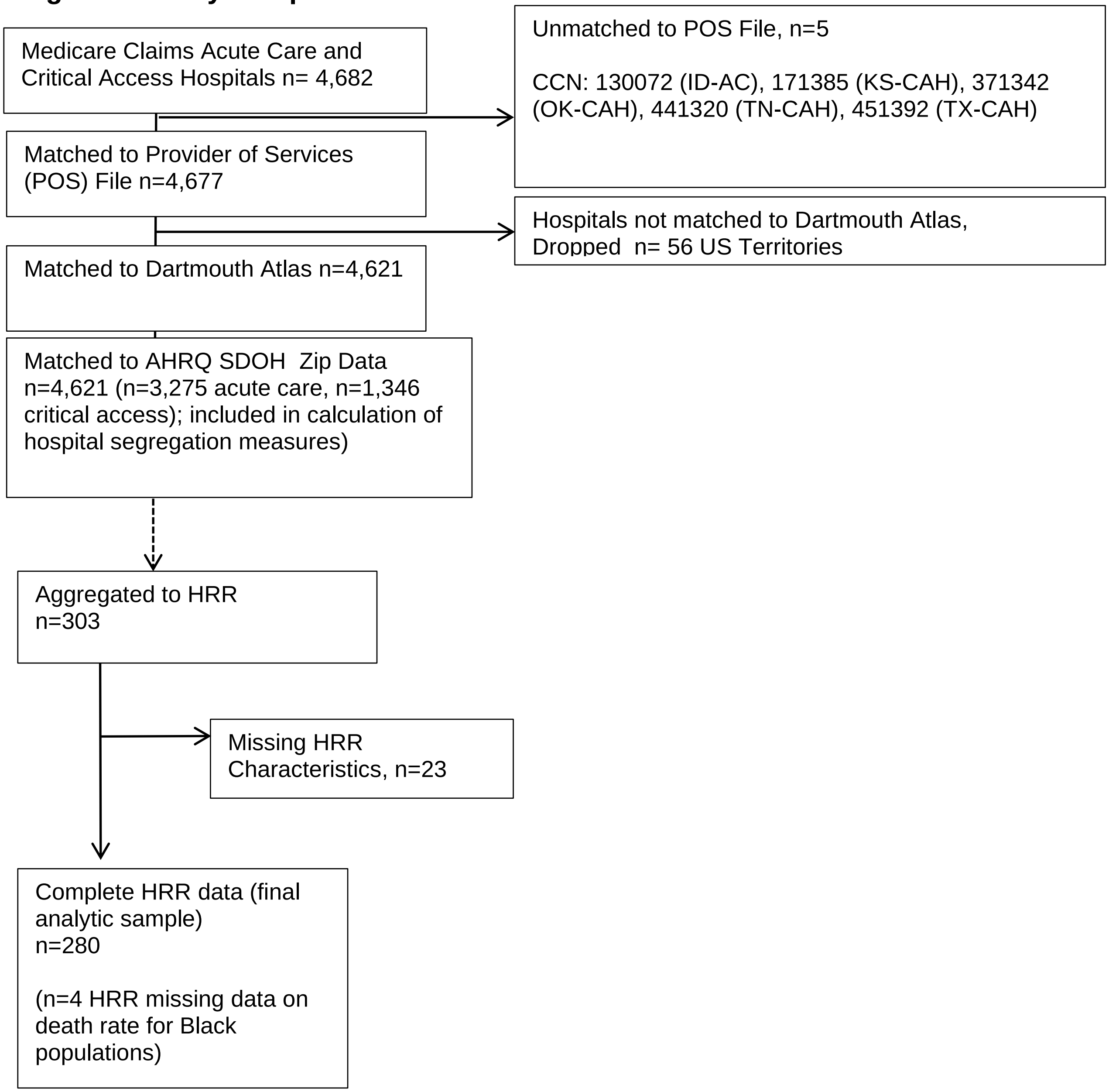

© 2023 Lin SC et al. *JAMA Health Forum.*

eFigure 3. Scatter Plots of Residential and Hospital Segregation Across US Hospital Referral Regions

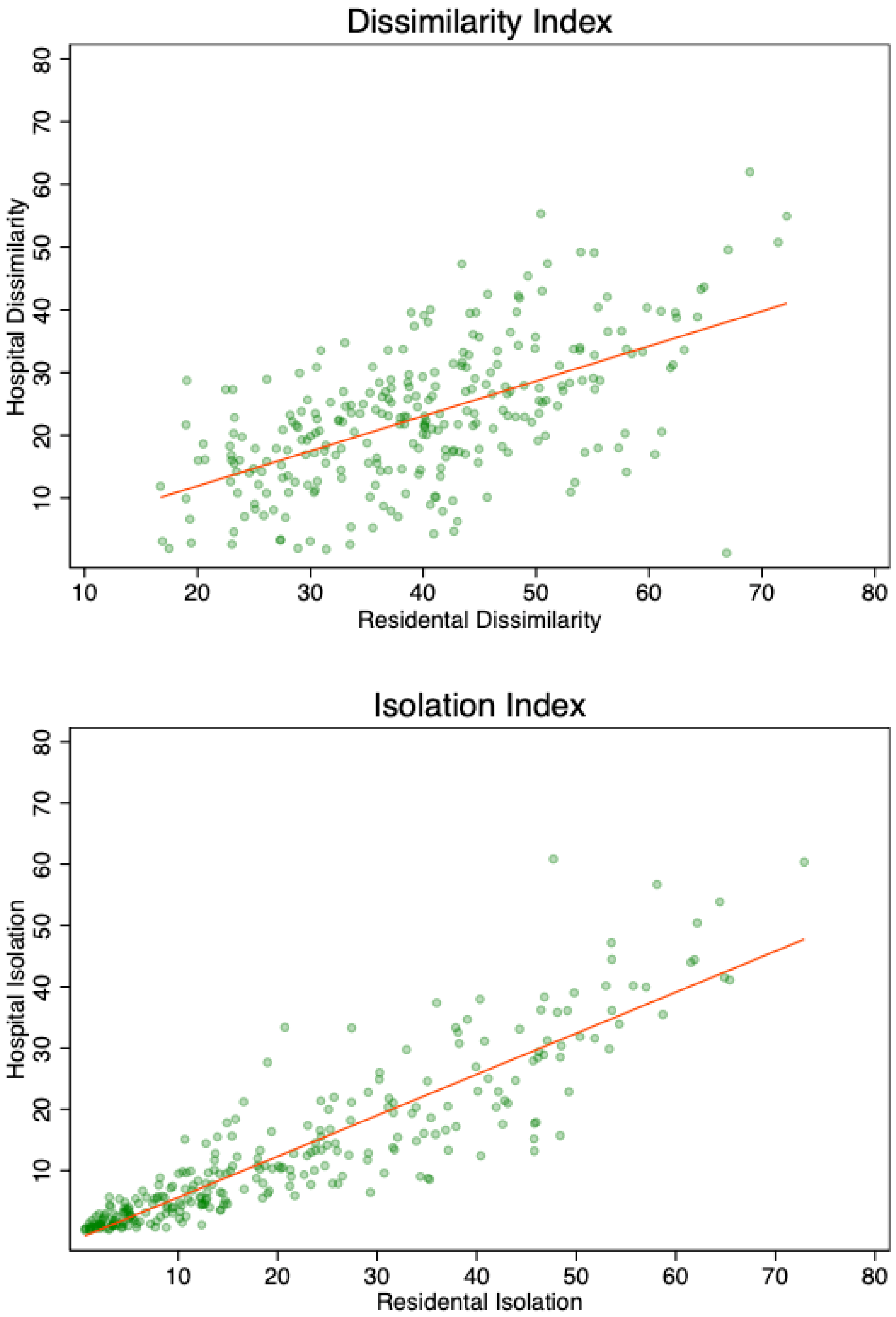

Notes: n=280 Hospital Referral Regions (HRR); Hospital segregation is calculated at the area (HRR) level to show how patterns of hospital visits are segregated by race between hospitals; Measures of hospital segregation are based on number of Medicare visits to area hospitals, residential segregation based on number of residents living in area zip-codes, lines represent linear trend

© 2023 Lin SC et al. *JAMA Health Forum.*

eTable 1. Correlation Matrix: Residential and Hospital Segregation

	Hospital Dissimilarity	Hospital Isolation	Residential Dissimilarity	Residential Isolation
Hospital Dissimilarity	1.00			
Hospital Isolation	0.26	1.00		
Residential Dissimilarity	0.58	0.27	1.00	
Residential Isolation	0.23	0.90	0.47	1.00

Notes: n=280 Hospital Referral Regions; Hospital segregation is calculated at the area (hospital referral region) level to show how patterns of hospital visits are segregated by race between hospitals

© 2023 Lin SC et al. *JAMA Health Forum.*

eTable 2. Fully Adjusted Regression Results: PQI Acute Composite

Prevention Quality Indicators Acute Composite per 100,000 Black Medicare Beneficiaries				
	**Hospital Dissimilarity**	**Hospital Isolation**	**Residential Dissimilarity**	**Residential Isolation**
**Hospital Segregation**				
Dissimilarity	-10 [-75,54]			
Isolation		43 [-83,169]		
**Residential Segregation**				
Dissimilarity			-49 [-111,12]	
Isolation				97 [-39,233]
**Community Covariates**				
% Non-Citizen	-21 [-43,0]	-22* [-44,-0]	-22 [-43,0]	-21 [-42,1]
Median Age	10 [-18,37]	8 [-20,36]	11 [-16,39]	7 [-20,35]
Median Income (1000's)	73 [-14,160]	68 [-18,154]	73 [-12,159]	67 [-19,152]
Diff in B/W Income (1000's)	2 [-56,60]	1 [-57,59]	-2 [-59,56]	2 [-55,60]
% Poverty Black	-3 [-15,8]	-2 [-14,9]	-3 [-14,8]	-2 [-14,9]
% Poverty White	8 [-15,31]	6 [-17,30]	8 [-15,31]	6 [-17,29]
% Less than HS Edu	24* [1,46]	24* [1,46]	22 [-0,45]	25* [2,47]
% Medicaid	-1 [-19,17]	-1 [-19,17]	1 [-17,19]	-3 [-20,15]
% Medicare	49 [-13,112]	53 [-10,115]	48 [-14,110]	49 [-13,111]
% Employer Insured	22 [-162,205]	34 [-150,218]	12 [-170,193]	36 [-146,219]
No. Hospitals	-3 [-9,3]	-4 [-9,2]	-2 [-8,3]	-4 [-10,1]
% Residents Black	16*** [10,21]	12* [0,24]	16*** [10,22]	8 [-4,20]
No. Residents (Millions)	16 [-24,55]	12 [-28,52]	16 [-23,56]	12 [-27,52]
% Population Urban	5* [1,8]	5* [1,8]	5** [1,9]	4* [0,8]
Region (Ref: Midwest)				
Northeast	-74 [-248,99]	-55 [-232,123]	-80 [-252,91]	-50 [-224,123]
South	-29 [-200,142]	-3 [-175,169]	-68 [-240,105]	19 [-153,192]
West	-147 [-329,35]	-125 [-307,56]	-207* [-400,-13]	-79 [-275,116]
Constant	-1049 [-2368,271]	-938 [-2280,404]	-1136 [-2452,179]	-809 [-2156,537]
N	280	280	280	280
r2_a	0.29	0.29	0.3	0.29

Notes: * p<0.05, **p<0.01, ***p<0.001

[Continued on next page]

© 2023 Lin SC et al. *JAMA Health Forum.*

Prevention Quality Indicators Acute Composite per 100,000 White Medicare Beneficiaries				
	Hospital Dissimilarity	Hospital Isolation	Residential Dissimilarity	Residential Isolation
**Hospital Segregation**				
Dissimilarity	-15 [-61,32]			
Isolation		-35 [-126,55]		
**Residential Segregation**				
Dissimilarity			-59** [-103,-15]	
Isolation				-60 [-158,38]
**Community Covariates**				
% Non-Citizen	-23** [-39,-7]	-23** [-38,-7]	-24** [-39,-8]	-24** [-39,-8]
Median Age	-16 [-35,4]	-15 [-35,5]	-13 [-33,6]	-15 [-35,5]
Median Income (1000's)	47 [-16,109]	46 [-16,108]	46 [-15,108]	46 [-16,108]
Diff in B/W Income (1000's)	-1 [-43,40]	0 [-42,41]	-6 [-47,36]	-2 [-43,40]
% Poverty Black	6 [-2,15]	6 [-2,15]	6 [-2,15]	6 [-2,15]
% Poverty White	0 [-17,16]	0 [-16,17]	0 [-17,16]	0 [-16,17]
% Less than HS Edu	21* [5,37]	21* [5,38]	20* [4,36]	21* [4,37]
% Medicaid	5 [-8,18]	5 [-8,17]	7 [-5,20]	6 [-7,18]
% Medicare	43 [-2,88]	41 [-5,86]	41 [-3,86]	43 [-2,88]
% Employer Insured	87 [-45,219]	84 [-49,217]	76 [-55,206]	84 [-48,216]
No. Hospitals	9*** [5,13]	9*** [5,13]	10*** [6,14]	9*** [5,13]
% Residents Black	3 [-2,7]	5 [-3,14]	3 [-1,7]	7 [-2,16]
No. Residents (Millions)	-45** [-74,-16]	-44** [-73,-15]	-44** [-72,-16]	-44** [-73,-16]
% Population Urban	-1 [-4,2]	-1 [-4,2]	-1 [-3,2]	-1 [-3,2]
Region (Ref: Midwest)				
Northeast	-5 [-130,121]	-11 [-139,117]	-11 [-134,112]	-11 [-136,115]
South	142* [18,266]	139* [14,263]	98 [-25,222]	129* [4,253]
West	-327*** [-458,-196]	-329*** [-460,-198]	-396*** [-535,-257]	-354*** [-496,-213]
Constant	785 [-168,1738]	733 [-236,1703]	685 [-257,1627]	673 [-301,1647]
N	280	280	280	280
r2_a	0.45	0.45	0.46	0.45

Notes: * p<0.05, **p<0.01, ***p<0.001

[Continued on Next Page]

© 2023 Lin SC et al. *JAMA Health Forum.*

Racial Difference: Prevention Quality Indicators Acute Composite per 100,000 Medicare Beneficiaries				
	**Hospital Dissimilarity**	**Hospital Isolation**	**Residential Dissimilarity**	**Residential Isolation**
**Hospital Segregation**				
Dissimilarity	5 [-59,69]			
Isolation		78 [-46,203]		
**Residential Segregation**				
Dissimilarity			10 [-51,70]	
Isolation				78 [-46,203]
**Community Covariates**				
% Non-Citizen	2 [-20,23]	0 [-21,22]	2 [-20,23]	0 [-21,22]
Median Age	25 [-2,52]	23 [-4,50]	25 [-2,52]	23 [-4,50]
Median Income (1000's)	26 [-60,112]	22 [-64,107]	27 [-58,112]	22 [-64,107]
Diff in B/W Income (1000's)	3 [-54,61]	1 [-56,58]	4 [-54,61]	1 [-56,58]
% Poverty Black	-10 [-21,2]	-9 [-20,3]	-10 [-21,2]	-9 [-20,3]
% Poverty White	9 [-14,32]	6 [-17,29]	9 [-14,32]	6 [-17,29]
% Less than HS Edu	3 [-20,25]	2 [-20,24]	3 [-20,25]	2 [-20,24]
% Medicaid	-6 [-24,12]	-6 [-23,12]	-6 [-24,11]	-6 [-23,12]
% Medicare	7 [-55,69]	12 [-50,74]	7 [-55,69]	12 [-50,74]
% Employer Insured	-65 [-247,117]	-50 [-232,133]	-64 [-246,117]	-50 [-232,133]
No. Hospitals	-12*** [-18,-6]	-12*** [-18,-7]	-12*** [-18,-7]	-12*** [-18,-7]
% Residents Black	13*** [7,19]	7 [-5,18]	13*** [7,19]	7 [-5,18]
No. Residents (Millions)	60** [21,100]	56** [16,96]	60** [21,100]	56** [16,96]
% Population Urban	6** [2,10]	6** [2,9]	6** [2,10]	6** [2,9]
Region (Ref: Midwest)				
Northeast	-70 [-242,103]	-44 [-219,132]	-70 [-240,101]	-44 [-219,132]
South	-171* [-341,-2]	-142 [-312,29]	-166 [-338,6]	-142 [-312,29]
West	180 [-1,360]	204* [24,384]	189 [-4,382]	204* [24,384]
Constant	-1834** [-3143,-524]	-1672* [-3001,-342]	-1821** [-3133,-510]	-1672* [-3001,-342]
N	280	280	280	280
r2_a	0.36	0.37	0.36	0.37

Notes: n=280 Hospital Referral Regions; * p<0.05, **p<0.01, ***p<0.001 ; Hospital segregation is calculated at the area (HRR) level to show how patterns of hospital visits are segregated by race between hospitals

© 2023 Lin SC et al. *JAMA Health Forum.*

eTable 3. Fully Adjusted Regression Results: PQI Chronic Composite

Prevention Quality Indicators Chronic Composite per 100,000 Black Medicare Beneficiaries				
	**Hospital Dissimilarity**	**Hospital Isolation**	**Residential Dissimilarity**	**Residential Isolation**
**Hospital Segregation**				
Dissimilarity	107 [-143,358]			
Isolation		383 [-104,871]		
**Residential Segregation**				
Dissimilarity			131 [-107,370]	
Isolation				510 [-18,1037]
**Community Covariates**				
% Non-Citizen	54 [-31,139]	48 [-37,133]	55 [-30,140]	58 [-27,142]
Median Age	44 [-62,151]	36 [-71,143]	41 [-65,148]	36 [-70,143]
Median Income (1000's)	-188 [-526,149]	-193 [-528,141]	-172 [-506,161]	-188 [-520,145]
Diff in B/W Income (1000's)	287* [62,511]	277* [52,501]	296* [70,521]	290* [66,514]
% Poverty Black	4 [-41,48]	7 [-38,51]	2 [-42,46]	5 [-39,49]
% Poverty White	26 [-63,116]	16 [-75,107]	29 [-60,118]	21 [-68,110]
% Less than HS Edu	30 [-58,118]	26 [-61,114]	32 [-56,120]	34 [-54,122]
% Medicaid	0 [-69,69]	3 [-66,72]	-4 [-73,66]	-5 [-74,63]
% Medicare	-15 [-257,227]	8 [-235,252]	-14 [-256,228]	-20 [-261,221]
% Employer Insured	-398 [-1111,314]	-348 [-1062,366]	-396 [-1107,314]	-370 [-1077,337]
No. Hospitals	-16 [-39,6]	-14 [-35,8]	-16 [-38,6]	-18 [-40,4]
% Residents Black	26* [3,48]	-4 [-49,41]	25* [2,47]	-13 [-59,34]
No. Residents (Millions)	72 [-82,227]	56 [-101,212]	76 [-78,229]	65 [-89,219]
% Population Urban	25*** [11,40]	24** [10,39]	24** [10,39]	22** [7,37]
Region (Ref: Midwest)				
Northeast	-450 [-1126,227]	-360 [-1047,328]	-469 [-1137,200]	-393 [-1065,280]
South	-487 [-1152,179]	-408 [-1074,259]	-448 [-1121,225]	-357 [-1027,312]
West	-1475*** [-2181,-768]	-1414*** [-2119,-710]	-1371*** [-2127,-615]	-1226** [-1985,-468]
Constant	1087 [-4045,6219]	1737 [-3469,6943]	1184 [-3950,6319]	2073 [-3154,7300]
N	280	280	280	280
r2_a	0.27	0.27	0.27	0.28

[Continued on Next Page]

© 2023 Lin SC et al. *JAMA Health Forum.*

Prevention Quality Indicators Acute Composite per 100,000 White Medicare Beneficiaries				
	**Hospital Dissimilarity**	**Hospital Isolation**	**Residential Dissimilarity**	**Residential Isolation**
**Hospital Segregation**				
Dissimilarity	35 [-40,109]			
Isolation		133 [-12,278]		
**Residential Segregation**				
Dissimilarity			-3 [-74,69]	
Isolation				143 [-14,301]
**Community Covariates**				
% Non-Citizen	-53*** [-78,-27]	-55*** [-80,-30]	-53*** [-78,-28]	-52*** [-77,-27]
Median Age	23 [-9,55]	20 [-12,52]	24 [-8,56]	21 [-11,53]
Median Income (1000's)	125* [25,226]	123* [24,222]	133** [33,232]	126* [27,225]
Diff in B/W Income (1000's)	-52 [-119,15]	-56 [-123,11]	-53 [-120,15]	-51 [-118,15]
% Poverty Black	7 [-7,20]	8 [-5,21]	6 [-7,19]	7 [-6,20]
% Poverty White	43** [16,69]	39** [12,66]	44** [17,70]	41** [15,68]
% Less than HS Edu	36** [10,63]	35** [9,62]	36** [10,62]	38** [12,64]
% Medicaid	17 [-4,37]	17 [-3,38]	17 [-4,38]	15 [-6,36]
% Medicare	-20 [-92,52]	-12 [-85,60]	-21 [-94,51]	-22 [-94,50]
% Employer Insured	-123 [-335,89]	-105 [-317,108]	-134 [-346,78]	-116 [-327,94]
No. Hospitals	-2 [-9,5]	-1 [-8,5]	-1 [-8,5]	-2 [-9,4]
% Residents Black	3 [-4,10]	-7 [-21,6]	3 [-3,10]	-8 [-21,6]
No. Residents (Millions)	20 [-26,66]	14 [-32,61]	23 [-23,69]	19 [-27,64]
% Population Urban	6* [1,10]	5* [1,9]	5* [1,10]	5* [0,9]
Region (Ref: Midwest)				
Northeast	-111 [-312,90]	-78 [-283,126]	-127 [-326,73]	-97 [-297,104]
South	44 [-154,242]	74 [-124,272]	15 [-186,216]	77 [-122,277]
West	-877*** [-1087,-667]	-854*** [-1063,-644]	-904*** [-1129,-678]	-810*** [-1036,-584]
Constant	-195 [-1723,1333]	36 [-1512,1584]	-258 [-1791,1274]	75 [-1482,1633]
N	280	280	280	280
r2_a	0.49	0.49	0.49	0.49

[Continued on Next Page]

© 2023 Lin SC et al. *JAMA Health Forum.*

Racial Difference: Prevention Quality Indicators Acute Composite per 100,000 White Medicare Beneficiaries				
	Hospital Dissimilarity	Hospital Isolation	Residential Dissimilarity	Residential Isolation
**Hospital Segregation**				
Dissimilarity	73 [-156,301]			
Isolation		250 [-195,695]		
**Residential Segregation**				
Dissimilarity			134 [-83,351]	
Isolation				250 [-195,695]
**Community Covariates**				
% Non-Citizen	107** [30,185]	103** [25,181]	108** [31,185]	103** [25,181]
Median Age	21 [-76,118]	16 [-82,113]	17 [-80,114]	16 [-82,113]
Median Income (1000's)	-314* [-621,-6]	-316* [-621,-11]	-305* [-608,-2]	-316* [-621,-11]
Diff in B/W Income (1000's)	339** [134,544]	332** [128,537]	348*** [143,553]	332** [128,537]
% Poverty Black	-3 [-43,37]	-1 [-42,39]	-4 [-44,36]	-1 [-42,39]
% Poverty White	-16 [-98,65]	-23 [-106,60]	-15 [-96,67]	-23 [-106,60]
% Less than HS Edu	-7 [-87,73]	-9 [-89,71]	-4 [-84,76]	-9 [-89,71]
% Medicaid	-16 [-79,47]	-14 [-77,48]	-21 [-84,42]	-14 [-77,48]
% Medicare	5 [-215,226]	20 [-202,243]	7 [-213,227]	20 [-202,243]
% Employer Insured	-276 [-924,373]	-244 [-894,407]	-262 [-908,384]	-244 [-894,407]
No. Hospitals	-14 [-35,7]	-12 [-32,7]	-15 [-35,5]	-12 [-32,7]
% Residents Black	22* [2,43]	3 [-38,44]	21* [1,42]	3 [-38,44]
No. Residents (Millions)	52 [-89,193]	41 [-101,184]	53 [-87,193]	41 [-101,184]
% Population Urban	20** [7,33]	19** [6,32]	19** [5,32]	19** [6,32]
Region (Ref: Midwest)				
Northeast	-339 [-954,277]	-281 [-908,346]	-342 [-950,266]	-281 [-908,346]
South	-531 [-1137,75]	-482 [-1090,126]	-462 [-1075,150]	-482 [-1090,126]
West	-598 [-1241,45]	-560 [-1203,82]	-468 [-1155,220]	-560 [-1203,82]
Constant	1282 [-3390,5954]	1701 [-3047,6449]	1442 [-3227,6112]	1701 [-3047,6449]
N	280	280	280	280
r2_a	0.19	0.2	0.2	0.2

Notes: n=280 Hospital Referral Regions; * p<0.05, **p<0.01, ***p<0.001 ; Hospital segregation is calculated at the area (HRR) level to show how patterns of hospital visits are segregated by race between hospitals

© 2023 Lin SC et al. *JAMA Health Forum.*

eTable 4. FULLY Adjusted Regression Results: Heart Disease or Stroke Deaths

Heart Disease or Stroke Deaths per 100,000 Black Residents				
	Hospital Dissimilarity	Hospital Isolation	Residential Dissimilarity	Residential Isolation
**Hospital Segregation**				
Dissimilarity	-3 [-8,1]			
Isolation		13** [5,21]		
**Residential Segregation**				
Dissimilarity			-1.1 [-5.3,3.0]	
Isolation				10.9* [1.9,19.9]
**Community Covariates**				
% Non-Citizen	-2** [-4,-1]	-2** [-4,-1]	-2.1** [-3.6,-0.7]	-2.0** [-3.5,-0.6]
Median Age	0 [-2,2]	-1 [-2,1]	-0.2 [-2.0,1.7]	-0.4 [-2.3,1.4]
Median Income (1000's)	7* [1,12]	5 [-1,11]	5.9* [0.1,11.8]	5.4 [-0.4,11.2]
Diff in B/W Income (1000's)	3 [-2,7]	2 [-2,7]	2.7 [-1.9,7.2]	2.9 [-1.6,7.4]
% Poverty Black	0 [-0,1]	1 [-0,1]	0.4 [-0.4,1.2]	0.5 [-0.4,1.3]
% Poverty White	2* [0,4]	1 [-0,3]	1.9* [0.3,3.5]	1.7* [0.2,3.3]
% Less than HS Edu	2** [1,4]	2** [0,3]	2.0** [0.5,3.5]	2.2** [0.7,3.7]
% Medicaid	0 [-1,1]	0 [-1,1]	0.2 [-1.0,1.4]	0 [-1.2,1.1]
% Medicare	1 [-3,6]	3 [-2,7]	1.5 [-2.6,5.7]	1.6 [-2.5,5.7]
% Employer Insured	-15* [-27,-3]	-11 [-23,1]	-14.3* [-26.4,-2.1]	-12.7* [-24.8,-0.7]
No. Hospitals	0 [-0,1]	0 [-0,0]	0.1 [-0.3,0.5]	0 [-0.4,0.4]
% Residents Black	0 [-0,1]	-1* [-2,-0]	0.1 [-0.3,0.5]	-0.7 [-1.5,0.0]
No. Residents (Millions)	1 [-2,3]	0 [-3,2]	0.6 [-2.1,3.2]	0.2 [-2.4,2.8]
% Population Urban	0 [-0,0]	0 [-0,0]	-0.1 [-0.4,0.1]	-0.2 [-0.5,0.1]
% Non-Citizen				
Northeast	-23*** [-34,-11]	-17** [-28,-5]	-21.5*** [-33.0,-10.0]	-18.9** [-30.4,-7.4]
South	-5 [-17,6]	3 [-8,14]	-3.9 [-15.5,7.6]	1.7 [-9.7,13.1]
West	-17** [-29,-4]	-10 [-22,2]	-15.9* [-29.1,-2.8]	-7.5 [-20.5,5.6]
Constant	37 [-51,125]	72 [-16,160]	40.1 [-48.4,128.6]	68.7 [-20.9,158.2]
N	276	276	276	276
r2_a	0.24	0.26	0.23	0.25

[Continued on Next Page]

© 2023 Lin SC et al. *JAMA Health Forum.*

Heart Disease or Stroke Deaths per 100,000 White Residents				
	Hospital Dissimilarity	Hospital Isolation	Residential Dissimilarity	Residential Isolation
**Hospital Segregation**				
Dissimilarity	-0.6 [-2.4,1.3]			
Isolation		0 [-3.6,3.6]		
**Residential Segregation**				
Dissimilarity			0.4 [-1.3,2.2]	
Isolation				4.1* [0.2,7.9]
**Community Covariates**				
% Non-Citizen	-1.0** [-1.6,-0.4]	-1.0** [-1.6,-0.4]	-1.0** [-1.6,-0.4]	-1.0** [-1.6,-0.4]
Median Age	-1.1** [-1.9,-0.3]	-1.1** [-1.9,-0.3]	-1.1** [-1.9,-0.3]	-1.2** [-2.0,-0.4]
Median Income (1000's)	0.2 [-2.3,2.7]	0.1 [-2.4,2.5]	0 [-2.4,2.5]	-0.1 [-2.6,2.3]
Diff in B/W Income (1000's)	0.2 [-1.5,1.8]	0.2 [-1.5,1.8]	0.2 [-1.5,1.8]	0.2 [-1.5,1.8]
% Poverty Black	-0.1 [-0.4,0.2]	-0.1 [-0.4,0.3]	-0.1 [-0.4,0.3]	0 [-0.4,0.3]
% Poverty White	0.8* [0.1,1.4]	0.8* [0.1,1.4]	0.8* [0.1,1.4]	0.7* [0.0,1.3]
% Less than HS Edu	0.9** [0.2,1.5]	0.9** [0.2,1.5]	0.9** [0.2,1.5]	0.9** [0.3,1.6]
% Medicaid	0.6* [0.0,1.1]	0.5* [0.0,1.1]	0.5* [0.0,1.0]	0.5 [-0.0,1.0]
% Medicare	2.2* [0.4,4.0]	2.2* [0.4,4.0]	2.2* [0.5,4.0]	2.2* [0.4,4.0]
% Employer Insured	-6.1* [-11.4,-0.9]	-5.9* [-11.2,-0.7]	-5.8* [-11.1,-0.6]	-5.5* [-10.7,-0.3]
No. Hospitals	0.1 [-0.1,0.3]	0.1 [-0.1,0.3]	0.1 [-0.1,0.2]	0.1 [-0.1,0.2]
% Residents Black	-0.2* [-0.4,-0.0]	-0.2 [-0.5,0.1]	-0.2* [-0.4,-0.0]	-0.5** [-0.8,-0.2]
No. Residents (Millions)	-0.1 [-1.2,1.1]	-0.1 [-1.3,1.0]	-0.1 [-1.3,1.0]	-0.2 [-1.4,0.9]
% Population Urban	-0.1 [-0.2,0.0]	-0.1 [-0.2,0.0]	-0.1 [-0.2,0.0]	-0.1 [-0.2,0.0]
Region (Ref: Midwest)				
Northeast	-6.1* [-11.1,-1.1]	-5.8* [-10.9,-0.7]	-5.7* [-10.7,-0.8]	-5.0* [-9.9,-0.1]
South	9.7*** [4.8,14.6]	10.2*** [5.2,15.1]	10.6*** [5.6,15.5]	11.9*** [7.0,16.8]
West	-9.7*** [-14.9,-4.4]	-9.3*** [-14.5,-4.1]	-8.7** [-14.3,-3.1]	-6.7* [-12.3,-1.1]
Constant	80.6*** [42.8,118.4]	81.5*** [43.0,120.0]	82.5*** [44.6,120.4]	90.9*** [52.4,129.3]
N	280	280	280	280
r2_a	0.58	0.58	0.58	0.59

[Continued on Next Page]

© 2023 Lin SC et al. *JAMA Health Forum.*

Racial Difference: Heart Disease or Stroke Deaths per 100,000 White Residents				
	Hospital Dissimilarity	Hospital Isolation	Residential Dissimilarity	Residential Isolation
**Hospital Segregation**				
Dissimilarity	-3 [-6,1]			
Isolation		13*** [6,20]		
**Residential Segregation**				
Dissimilarity			-2 [-5,2]	
Isolation				13*** [6,20]
**Community Covariates**				
% Non-Citizen	-1 [-2,0]	-1* [-3,-0]	-1 [-2,0]	-1* [-3,-0]
Median Age	1 [-1,3]	1 [-1,2]	1 [-1,3]	1 [-1,2]
Median Income (1000's)	6* [1,11]	5 [-0,10]	6* [1,11]	5 [-0,10]
Diff in B/W Income (1000's)	3 [-1,7]	2 [-1,6]	3 [-1,7]	2 [-1,6]
% Poverty Black	0 [-0,1]	1 [-0,1]	0 [-0,1]	1 [-0,1]
% Poverty White	1 [-0,3]	1 [-1,2]	1 [-0,2]	1 [-1,2]
% Less than HS Edu	1 [-0,3]	1 [-0,2]	1 [-0,3]	1 [-0,2]
% Medicaid	0 [-1,1]	0 [-1,1]	0 [-1,1]	0 [-1,1]
% Medicare	-1 [-4,3]	0 [-3,4]	-1 [-4,3]	0 [-3,4]
% Employer Insured	-9 [-19,2]	-5 [-15,5]	-8 [-19,2]	-5 [-15,5]
No. Hospitals	0 [-0,0]	0 [-0,0]	0 [-0,0]	0 [-0,0]
% Residents Black	0 [-0,1]	-1* [-1,-0]	0 [-0,1]	-1* [-1,-0]
No. Residents (Millions)	1 [-1,3]	0 [-2,2]	1 [-2,3]	0 [-2,2]
% Population Urban	0 [-0,0]	0 [-0,0]	0 [-0,0]	0 [-0,0]
Region (Ref: Midwest)				
Northeast	-16** [-26,-7]	-11* [-21,-1]	-16** [-26,-6]	-11* [-21,-1]
South	-15** [-25,-6]	-8 [-17,2]	-15** [-25,-5]	-8 [-17,2]
West	-7 [-18,3]	-1 [-11,9]	-8 [-19,4]	-1 [-11,9]
Constant	-45 [-120,30]	-11 [-86,64]	-44 [-120,32]	-11 [-86,64]
N	276	276	276	276
r2_a	0.07	0.11	0.07	0.11

Notes: n=280 Hospital Referral Regions; * p<0.05, **p<0.01, ***p<0.001 ; Hospital segregation is calculated at the area (HRR) level to show how patterns of hospital visits are segregated by race between hospitals

© 2023 Lin SC et al. *JAMA Health Forum.*

eTable 5. Sensitivity Analysis, Regional Random Effects

	Hospital Dissimilarity Mean National Effect (95% CI)		Hospital Isolation Mean National Effect (95% CI)	
Residential Dissimilarity	0.45***	[0.36,0.54]		
Residential Isolation			0.30***	[0.13,0.47]
% Non-Citizen	0.01	[-0.38,0.39]	0.23*	[0.01,0.44]
Median Age	0.09	[-0.40,0.57]	0.13	[-0.14,0.41]
Median Income (1000's)	2.12**	[0.63,3.62]	1.12	[-0.11,2.36]
Diff in B/W Income (1000's)	0.52	[-0.45,1.49]	0.35	[-0.37,1.06]
% Poverty Black	-0.17	[-0.37,0.02]	-0.07	[-0.18,0.03]
% Poverty White	0.32	[-0.08,0.71]	0.37**	[0.14,0.60]
% Less than HS Edu	-0.01	[-0.41,0.38]	0.08	[-0.14,0.30]
% Medicaid	-0.03	[-0.34,0.28]	-0.13	[-0.30,0.03]
% Medicare	-0.04	[-1.13,1.06]	-0.33	[-0.95,0.29]
% Employer Insured	-1.87	[-5.07,1.32]	-1.83*	[-3.61,-0.05]
No. Hospitals	0.28***	[0.19,0.38]	0	[-0.05,0.05]
% Residents Black	0.02	[-0.09,0.12]	0.80***	[0.65,0.95]
No. Residents (Millions)	0.19	[-0.44,0.81]	0.35	[-0.01,0.71]
% Population Urban	-0.06	[-0.12,0.00]	-0.02	[-0.05,0.02]
Region (Ref: Midwest)				
Northeast	-3.56*	[-6.56,-0.55]	7.93*	[1.31,14.56]
South	-3.64*	[-6.69,-0.60]	-2.84	[-8.84,3.15]
West	0.3	[-3.09,3.68]	7.48*	[1.18,13.78]
Constant	-6.57	[-29.24,16.10]	-13.98*	[-27.27,-0.68]
n	280		280	
R2	0.616		0.889	

Notes: n=280 Hospital Referral Regions; * p<0.05, **p<0.01, ***p<0.001 ; Hospital segregation is calculated at the area (HRR) level to show how patterns of hospital visits are segregated by race between hospitals

© 2023 Lin SC et al. *JAMA Health Forum.*

eTable 6. Sensitivity Analysis, Weighted by HRR population: Multiple Linear Regression Results, Predictors of Hospital Segregation

	Hospital Dissimilarity		Hospital Isolation	
Residential Dissimilarity	0.45***	[0.36,0.55]		
Residential Isolation			0.35***	[0.26,0.44]
% Non-Citizen	0.01	[-0.39,0.41]	0.25*	[0.00,0.49]
Median Age	0.07	[-0.43,0.57]	0.26	[-0.04,0.57]
Median Income (1000's)	2.04*	[0.48,3.60]	0.66	[-0.30,1.62]
Diff in B/W Income (1000's)	0.35	[-0.70,1.41]	0.37	[-0.28,1.02]
% Poverty Black	-0.18	[-0.39,0.03]	-0.13	[-0.25,0.00]
% Poverty White	0.35	[-0.06,0.77]	0.36**	[0.11,0.62]
% Less than HS Edu	0.02	[-0.39,0.44]	0.13	[-0.12,0.39]
% Medicaid	-0.07	[-0.40,0.25]	-0.12	[-0.32,0.08]
% Medicare	-0.13	[-1.26,1.00]	-0.90*	[-1.60,-0.21]
% Employer Insured	-1.99	[-5.31,1.34]	-2.05*	[-4.09,-0.01]
No. Hospitals	0.23***	[0.12,0.33]	-0.04	[-0.10,0.03]
% Residents Black	0.02	[-0.09,0.12]	0.56***	[0.43,0.70]
No. Residents (Millions)	0.57	[-0.15,1.29]	0.62**	[0.18,1.07]
% Population Urban	-0.08*	[-0.14,-0.01]	-0.01	[-0.05,0.03]
Region (Ref: Midwest)				
Northeast	-3.71*	[-6.84,-0.58]	-3.34***	[-5.28,-1.40]
South	-3.79*	[-6.94,-0.64]	-2.94**	[-4.87,-1.01]
West	-0.43	[-3.97,3.11]	-0.67	[-2.86,1.52]
Constant	-2.52	[-26.48,21.44]	-8.37	[-23.14,6.40]
n	280		280	
R2	0.616		0.889	

Notes: n=280 Hospital Referral Regions; * p<0.05, **p<0.01, ***p<0.001 ; Hospital segregation is calculated at the area (HRR) level to show how patterns of hospital visits are segregated by race between hospitals

© 2023 Lin SC et al. *JAMA Health Forum.*

eTable 7. Sensitivity Analysis, Weighted by HRR Resident Population: Standardized Associations Between Hospital Segregation and Health Outcomes

	Prevention Quality Indicators Acute Composite per 100,000 Medicare Beneficiaries		Prevention Quality Indicators Chronic Composite per 100,000 Medicare Beneficiaries		Heart Disease or Stroke Deaths per 100,000 Residents	
**HRR[1]-Level Median and Interquartile Range**						
Black populations	732 (368, 986)		4535 (3221, 5480)		98 (84, 113)	
White populations	1107 (905, 1348)		2632 (2132, 2973)		58 (47, 70)	
Difference	-392 (-691, -127)		1923 (1007, 2656)		8.9 (3.3, 18.5)	
**Regression Results (Coefficient (95% Confidence Intervals))**						
Hospital Dissimilarity	Unadjusted	Adjusted	Unadjusted	Adjusted	Unadjusted	Adjusted
Black Populations	-16 [-58,26]	**-49.2* [-98.0,-0.5]**	94 [-76,264]	-47.1 [-243.2,149.1]	-0.7 [-3.7,2.3]	-3.5 [-7.1,0.01]
White Populations	-21 [-59,17]	-14.3 [-53.3,24.8]	-30 [-96,36]	-21.4 [-85.9,43.1]	**-3.9*** [-5.7,-2.1]**	-0.7 [-2.3,0.9]
Difference	5 [-40,50]	-35 [-81.5,11.6]	124 [-20,267]	-25.7 [-198.4,147.0]	**3.3** [1.0,5.5]**	-2.8 [-5.8,0.2]
Hospital Isolation	Unadjusted	Adjusted	Unadjusted	Adjusted	Unadjusted	Adjusted
Black Populations	**147*** [112,182]**	12.5 [-78.4,103.3]	**573*** [431,715]**	147.7 [-214.9,510.3]	**5.0*** [2.3,7.7]**	**12.1*** [5.7,18.6]**
White Populations	**36* [2,71]**	**-80.9* [-152.4,-9.3]**	**121*** [61,181]**	-14.8 [-134.2,104.6]	0.2 [-1.5,1.9]	1.0 [-2.0,4.0]
Difference	**111*** [72,150]**	**93.3* [7.6,179.0]**	**452*** [330,574]**	162.5 [-156.5,481.5]	**4.9*** [2.8,6.9]**	**11.0*** [5.6,16.4]**
Residential Dissimilarity	Unadjusted	Adjusted	Unadjusted	Adjusted	Unadjusted	Adjusted
Black Populations	20 [-24,63]	**-61.9* [-113.8,-9.9]**	**342*** [169,515]**	60.5 [-148.9,270.0]	1.2 [-1.9,4.3]	0.0 [-3.8,3.8]
White Populations	37 [-2,76]	**-49.6* [-90.9,-8.4]**	**175*** [109,241]**	-20.4 [-89.3,48.5]	-1.4 [-3.3,0.5]	0.2 [-1.5,2.0]
Difference	-18 [-64,29]	-12.2 [-62.1,37.7]	**167* [18,316]**	80.9 [-103.4,265.1]	**2.6* [0.3,5.0]**	-0.2 [-3.4,3.0]
Residential Isolation	Unadjusted	Adjusted	Unadjusted	Adjusted	Unadjusted	Adjusted
Black Populations	**163*** [126,200]**	48.3 [-57.1,153.8]	**635*** [484,785]**	416 [-2.8,834.9]	**4.2** [1.3,7.1]**	**10.0** [2.4,17.6]**
White Populations	**99*** [64,135]**	-38.3 [-122.2,45.5]	**242*** [183,301]**	97.3 [-41.0,235.6]	1.7 [-0.1,3.5]	**3.8* [0.3,7.3]**
Difference	**64** [20,108]**	**93.3* [7.6,179.0]**	**393*** [258,527]**	162.5 [-156.5,481.5]	**2.6* [0.4,4.9]**	**11.0*** [5.6,16.4]**

Notes: * p<0.05, **p<0.01, ***p<0.001; [1]HRR=Hospital Referral Region; [2]Adjusted models include: % non citizen, median age, median income, racial differences in income, % poverty black, % poverty white, % less than high school educated, % Medicaid, % Medicare, % Employee insured, number of hospitals, % residents Black, number of residents, % population urban, and geographic region; significant effects in bold; Hospital segregation is calculated at the area (HRR) level to show how patterns of hospital visits are segregated by race between hospitals

© 2023 Lin SC et al. *JAMA Health Forum.*
