# Supplementary material for: Segregated Patterns of Hospital Care Delivery and Health Outcomes
Source: JAMA Health Forum. 2023 Nov 22;4(11):e234172. doi: 10.1001/jamahealthforum.2023.4172 (PMC10665978; doi:10.1001/jamahealthforum.2023.4172)
Supplement: Supplement 2. — Data Sharing Statement [file jamahealthforum-e234172-s002.pdf]

Data Sharing Statement

Lin. Segregated Patterns of Hospital Care Delivery and Health Outcomes. *JAMA Health Forum*. Published November 22, 2023. doi:10.1001/jamahealthforum.2023.4172

Data
Data available: No

Additional Information
Explanation for why data not available: Data obtained under strict DUA
